# Is Racial Discrimination Associated with Number of Missing Teeth Among American Adults?

**DOI:** 10.1007/s40615-020-00891-8

**Published:** 2020-10-13

**Authors:** Malini Muralikrishnan, Wael Sabbah

**Affiliations:** Faculty of Dentistry, Oral & Craniofacial Sciences, King’s College London, 2nd Floor Dental Extension, Bessemer Road, Denmark Hill, London, SE5 9RS UK

**Keywords:** Ethnicity, Oral health, Racial discrimination, Socioeconomic factors, Tooth loss

## Abstract

**Objective:**

The objectives of this study are to assess the association of racial discrimination with tooth loss among American adults and whether this relationship, if existed, explains ethnic differences in tooth loss.

**Methods:**

Data is from the Behavioural Risk Factor Surveillance System (BRFSS) 2014, a cross-sectional survey of a nationally representative sample of American adults. The survey included data on sociodemographic characteristics, behaviour, health insurance and number of missing teeth. The survey also included questions on whether a person was treated differently because of his/her race. Logistic regression analysis was conducted to assess the relationship between tooth loss and indicators of discrimination. We also examined the relation between ethnicity and indicators of discrimination.

**Results:**

The analysis included 4858 participants aged 18 to 44 years. Tooth loss (> one tooth) was reported by 26% of participants. Among those reporting discrimination at healthcare facility, there was 141% increase in tooth loss compared to those not reporting discrimination. Discrimination at work and emotional impact of discrimination were both significantly associated with tooth loss in the partially adjusted models. Accounting for discrimination slightly attenuated ethnic differences in too loss. Black Americans had significantly higher odds for reporting all types of discrimination used here.

**Conclusion:**

This study demonstrated a potential role for discrimination in tooth loss among American adults. Discrimination could also explain part of ethnic inequalities in oral health.

## Introduction

Despite the observed improvement in oral health over the past few decades, oral health inequalities continue to exist among those at the bottom of socioeconomic hierarchy and those living in more deprived and underserved areas [1, 2]. Inequalities in oral health have also been repeatedly demonstrated through ethnic and racial variations in different indicators of oral health [1, 3, 4]. It is worth noting that in the USA, White and Black/African Americans are recognised as race, while Hispanic American is an ethnicity [5]; hence, most of surveys in the USA use the term race/ethnicity to describe these groups. Several studies in the USA postulated that African and Hispanic Americans have fewer teeth, higher levels of dental caries and periodontal diseases and poorer oral health-related quality of life [6–10]. While racial/ethnic inequalities in oral health have been attributed to biological, behavioural and cultural factors, others have argued that socioeconomic factors could explain racial/ ethnic variations in oral health and related behaviours [4, 6, 10]. In other words, racial/ ethnic minorities are more likely to be subjected to adverse socioeconomic conditions, which impact their abilities to adopt healthier lifestyle, affect their psychological wellbeing and subsequently affect their general and oral health outcomes [10, 11].

On the other hand, racial/ ethnic discrimination also impacts opportunities for education, better employment and selection of living areas [12, 13]. Aside from the impact on adverse socioeconomic conditions, discrimination could have long and lasting effects on psychological wellbeing which persist even after their actual experience has come to an end [14, 15]. In other words, racial discrimination is linked to poorer health through materialistic, psychosocial, physiological and behavioural pathways [16].

While discrimination appears to impact psychological wellbeing [11, 17], it is plausible that it impacts certain oral conditions and oral health–related behaviours [18]. Furthermore, the association of discrimination with use of dental services was also demonstrated in the American population, where those experiencing emotional impact of discrimination were less likely to visit a dentist [19]. However, the association between racial/ethnic discrimination and oral health outcomes has not been adequately addressed in the literature. In this study, we set out to test the association between three indicators of racial discrimination, namely discrimination at healthcare facility, at work or the emotional impact of discrimination and oral health indicated by tooth loss. The objective of this study is to assess whether racial discrimination is independently associated with tooth loss among US adults aged 18 to 44 years, and whether this relationship, if existed, explains ethnic differences in tooth loss.

## Methods

### Data Source and Study Population

This study used data from the Behavioural Risk Factor Surveillance System (BRFSS) 2014, a nationally representative survey of non-institutionalised American adults.

Telephone interview which involved both cellphone and landline interviews were used for data collection. The 2014 survey was selected because it is the latest available survey that has information pertaining to discrimination.

The materials for training the interviewer were developed by the Centers for Disease Control and Prevention. Interviewers’ performances were monitored using surveillance sites. The survey included questions on racial discrimination, missing teeth, ethnicity, family income, education, type of insurance and dental visits.

### Outcome Variable

The outcome of interest is missing permanent teeth removed due to dental caries, gum disease or infection. Teeth lost due to injury or orthodontics treatment were excluded. The original question about tooth loss had 5 answers: ‘none’, ‘1–5’, ‘6 or more but not all’, ‘all’ and ‘do not know’. Given the relatively young age of the included sample where tooth loss is uncommon, the variable for tooth loss was categorised to indicate missing one or more tooth versus none. Those who answered ‘do not know’ were excluded from the analysis.

### Explanatory Variable

The main explanatory variable is racial discrimination. The item from the questionnaire used to assess this was taken from the module that has race in it. In the first two questions, participants were asked whether they were treated at work (question 1) or while seeking healthcare (question 2) worse, same or better than those from other races. These questions were dichotomised into worse versus better, same or did not encounter people from different race. Those reporting do not know or did not use healthcare services were excluded. The third question asked whether participants felt emotionally upset as a result of how they were treated because of their race. The answers for this question were yes, no, do know. Persons reporting do not know were excluded. These three different variables were used to indicate: discrimination at healthcare facility, at work and emotional impact of discrimination.

### Covariates

The selection of covariates included in the analysis was based on their known relationship with tooth loss [20, 21]. Demographic variables included age and sex. Age was reported in three groups:18–24, 25–34 and 35–44 years. Five groups of income were reported: lowest (less than US$15,000), second lowest (US$15,000 to less than 25,000) middle (US$25,000 to less than 35,000), second highest (US$35,000 to less than 50,000) and highest (US$50,000 or more). Education was categorised three groups to create meaningful categories. These were less than high school, high school, some college but no degree and college graduate. Race/ethnicity was reported in 4 groups White, Black, Hispanic, Asian/other and Multiracial. Self-rated general health was categorised into good/excellent and poor/fair. Variables indicating availability of different health insurance were combined to indicate availability of any health insurance. Dental visit was categorised to indicate any visit within last year versus less often or no visit. Diabetes was coded into diabetic versus no diabetic. Smoking was categorised into 3 groups: current smoker, former smoker and never smoked.

### Data Analysis

Stata 16 (StataCorp, College station, TX, USA) [22] was used to perform analysis. Sampling weights and survey commands were used throughout the analysis. Given that the outcome was missing one tooth or more, the analysis only included adults aged 18 to 44 years of age.

Descriptive analysis of all variables included in the analysis was conducted, namely missing teeth, discrimination in healthcare facilities, discrimination at work, emotional impact of discrimination, age, sex, race, annual income, education, self-rated general health, dental visits, health insurance, diabetes and smoking. The distributions of tooth loss (one or more tooth) within all included variables were assessed.

Logistic regression models were constructed to assess the relationship between tooth loss (one or more tooth) and discrimination in healthcare facility. The first model was adjusted for discrimination in healthcare, age and sex. Two additional models were constructed to assess whether discrimination in healthcare facilities attenuates ethnic inequalities in tooth loss. The first model was adjusted for age, sex, ethnicity, annual income, education, self-rated general health, dental visits, health insurance, diabetes and smoking. The second model was additionally adjusted for discrimination in healthcare facilities.

Two logistic regression models were constructed to assess the relationship between tooth loss and discrimination at work adjusting for age and sex. The second model was additionally adjusted for race, annual income, education, health insurance, dental visits, self-reported health, diabetes and smoking. Similar models were constructed for emotional impact of discrimination.

Finally, the relationship between the three indicators of discrimination and race was assessed using logistic regression and adjusting for race, age, sex, education and annual income.

## Results

Eligible participants who were included in the race module, had the question on number of teeth and those aged 18–44 were 5852. After excluding cases with missing values in any of the variables included in the analysis, the sample dropped to 4858 participants (17% missing from those eligible for the analysis).

Table 1 shows descriptive analysis of all the variables in this study and the distribution of tooth loss among all variables. Overall, 26% (95% confidence intervals ‘CI’ 24.3, 27.6) of participants reported missing one or more tooth. Discrimination at healthcare facilities, at work and emotional impact of discrimination were reported by 2.9%, 4.7% and 5.4%, respectively. Tooth loss was higher among those reporting discriminations.

**Table 1:** Distribution of variables and percentage of tooth loss among American adults aged 18–44, BRFSS 2014, *N* = 4858

Variables		Percentage (95% CI)	Percentage missing 1+ tooth within groups (95% CI)	Sig*
Sex	Male	53.2 (51.3, 55.1)	26.8 (24.4, 29.4)	NS
	Female	46.8 (45, 48.7)	25 (22.7, 27.2)	
Age groups	18–24	19.2 (17.7, 20.9)	11.9 (08.9, 15.6)	< 0.001
	25–34	40.7(38.8, 42.6)	26.3 (23.6, 29.1)	
	35–44	40.1(38.3, 41.9)	32.3 (26.7, 35)	
Race/ethnicity	White	64.4 (62.6, 66.2)	19.5 (17.8, 21.3)	< 0.001
	Black	14.6 (13.3, 16.1)	44 (37.7, 50.1)	
	Hispanic	13.1 (12.1, 14.2)	35.6 (30.8, 40.8)	
	Asian/Other	6.6 (5.8, 7.6)	31.7 (25.4, 38.9)	
	Multiracial	1.2 (0.8, 1.7)	15.3 (6.2, 32.8)	
Education	< High school	9.8 (8.4, 11.3)	46.1 (38.4, 54)	< 0.001
	High school	24 (22.4, 26)	32.6 (29, 36.4)	
	Some college no degree	35.3(33.5, 37.2)	25.9 (23, 29)	
	College graduate	30.9 (29.4,32.4)	14.3 (12.6, 16.2)	
Annual income	Lowest	7.3 (6.3, 8.5)	32.2 (25.4, 39.7)	< 0.001
	2nd lowest	18.3 (16.8, 20)	38 (33.2, 43)	
	Middle	12.1 (10.9, 13.5)	27.4 (22.6, 32.7)	
	2nd highest	15.2 (13.9, 16.6)	27.6 (23.5, 32.2)	
	Highest	47.1 (45.3, 48.9)	19.3 (17.3, 21.4)	
Tooth loss (1 or more)	No	74.1 (72.4, 75.8)		
	Yes	26 (24.3, 27.6)		
Health insurance	No	15 (13.4, 16.5)	37.1 (31.8, 43)	< 0.001
	Yes	85.1(83.5, 86.6)	23.9 (22.3, 25.7)	
Dental visit within past year	No	34.9 (33.1, 36.7)	28.7 (25.7, 31.8)	< 0.05
	Yes	65.1 (63.3, 67)	24.4 (22.5, 26.5)	
Discrimination in healthcare	No	97.1 (96.4, 97.7)	25.1 (23.4, 26.8)	< 0.001
	Yes	2.9 (2.3, 3.6)	53.3 (42.5, 63.8)	
Emotional impact of discrimination	No	95 (93.7, 95.4)	25.3 (23.6, 27.1)	< 0.01
	Yes	5.4 (4.6, 6.3)	36.2 (28.7, 44.4)	
Discrimination at work	No	95.3 (94.4, 96.1)	25.2 (23.5, 27)	< 0.001
	Yes	4.7 (3.9, 5.6)	40 (31.4, 49.3)	
Self-rated general health	Good/excellent	92.3 (91.2, 93.2)	24.6 (22.9, 26.4)	< 0.001
	Poor/fair	7.7 (6.8, 8.8)	41.2 (34.6, 48.2)	
Diabetes	Non-diabetic	97.3 (96.6, 97.9)	25.7 (24, 27.4)	NS
	Diabetic	2.7 (2.1, 3.4)	33.7(24.2, 44.7)	
Smoking	Current	21.8 (20.1, 23.5)	39.2 (35, 43.6)	< 0.001
	Former	17.7 (16.4, 19.1)	25.7 (22, 29.8)	
	Never	60.5 (58.7, 62.4)	21.2 (19.2, 23.3)	

*p value from chi square. *NS*, not significant

Table 2 shows the association between discrimination at healthcare facility and tooth loss. Those who experienced discrimination at healthcare facilities had a 141% increase in tooth loss compared to those who did not experience discrimination.

**Table 2:** Logistic regression analysis showing odds ratio and 95% CI for factors associated with tooth loss among American adults aged 18–44 years, BRFSS 2014, *N* = 4858

Variables		OR (95% CI)		
		Model 1	Model 2	Model 3
Sex (female)		0.90^NS^ (0.75, 1.07)	0.93^NS^ (0.77, 1.13)	0.94^NS^ (0.77, 1.93)
Age (references, 18–24)	25–34	2.66^***^ (1.88, 3.78)	3.15^***^ (2.19, 4.51)	3.11^***^ (2.17, 4.47)
	35–44	3.53^***^ (2.51, 4.97)	5.18^***^ (3.62, 7.41)	5.06^***^ (3.54, 7.23)
Experienced discrimination in healthcare facilities (reference: no experience of discrimination)		3.34 ^***^ (2.12, 5.26)		2.41^**^ (1.47, 3.98)
Race (reference, White)	Black		2.67^***^ (1.95, 3.66)	2.61^***^ (1.90, 3.59)
	Hispanic		1.80^***^ (1.34, 2.44)	1.76^***^ (1.30, 2.38)
	Asian/Other		2.04^***^ (1.42, 2.92)	2.02^***^ (1.41, 2.91)
	Multiracial		0.82^NS^ (0.27, 2.55)	0.76^NS^ (0.28, 2.08)
Annual income reference: lowest	2nd lowest		1.19^NS^ (0.78, 1.82)	1.19^NS^ (0.79, 1.81)
	Middle		0.79^NS^ (0.50, 1.26)	0.80^NS^ (0.51, 1.27)
	2nd highest		0.98^NS^ (0.63, 1.50)	0.98^NS^ (0.64, 1.50)
	Highest		0.69^NS^ (0.45, 1.06)	0.72^NS^ (0.47, 1.09)
Education level (reference: < high school)	High school		0.70^NS^ (0.46, 1.05)	0.69^NS^ (0.46, 1.04)
	Some college no degree		0.54^**^ (0.36, 0.81)	0.52^**^ (0.35, 0.79)
	College graduate		0.29^***^ (0.19, 0.45)	0.29^***^ (0.19, 0.44)
Having any health insurance (reference: no insurance)			1.00^NS^ (0.74, 1.36)	1.01^NS^ (0.75, 1.37)
Visited a dentist past year (reference: less often than past year/ never)			1.19^NS^ (0.96, 1.48)	1.19^NS^ (0.96, 1.48)
Self-rated health (fair/poor) (reference good/excellent)			1.48^*^ (1.07, 2.05)	1.43^*^ (1.03, 1.98)
Diabetic (reference non-diabetic)			0.94^NS^ (0.5, 1.72)	0.97^NS^ (0.53, 1.76)
Smoking (reference, current smoker)	Former		0.61^**^ (0.46, 0.81)	0.61^**^ (0.46, 0.80)
	Never		0.47^***^ (0.37, 0.61)	0.47^***^ (0.37, 0.60)

^***^p < 0.001, ^**^p < 0.01, ^*^p < 0.05, ^*NS*^, not significant

Model 1, adjusted for discrimination in healthcare, age and sex

Model 2, adjusted for age, gender, race, income, education, health insurance, dental visits, self-reported health, diabetes and smoking

Model 3, model 2+ discrimination at healthcare facilities

Model 2 has all the variables except discrimination and was assessed for tooth loss.

The odds of tooth loss among Blacks was 2.67 (95% CI 1.95, 3.66) compared to Whites. Among Hispanics, the odds ratio of tooth loss was 1.80 (95% CI 1.34, 2.44). After adjusting for discrimination at healthcare, the odds ratios for tooth loss by Blacks and Hispanics were slightly attenuated but remained significant. The odds for tooth loss among those reporting discriminations at healthcare in the fully adjusted model were 2.41 (95% CI 1.47, 3.98).

The odds for tooth loss among people who reported discrimination at work or had emotional impact of discriminations were 2.05 (95% CI 1.38, 3.03) (105% increase) and 1.62 (95% CI 1.14, 2.31) (62% increase), respectively. After adjusting for all covariates, these significant associations disappeared (Table 3). Race/ethnicity was significantly associated with all indicators of discrimination (Table 4).

**Table 3:** Logistic regression analysis showing odds ratio and 95% CI for tooth loss by discrimination at work and emotional impact of discrimination among American adults aged 18–44 years, BRFSS 2014, *N* = 4858

	OR (95% CI)	
	Model 1	Model 2
Experienced discrimination at work (reference, no discrimination at work)	2.05^***^ (1.38, 3.03)	1.21^NS^ (0.73, 2.00)
Had emotional impact of discrimination (reference, no impact of discrimination)	1.62^**^ (1.14, 2.31)	0.98^NS^ (0.63, 1.53)

^***^p < 0.001, ^**^p < 0.01, ^*NS*^, not significant

Model 1 adjusted for discrimination variable, sex and age

Model2 additionally adjusted for race, income, education, health insurance, dental visits, self-reported health, diabetes and smoking

**Table 4:** Logistic regression analysis showing odds ratio and 95% CI showing association between race/ethnicity and discrimination among American adults aged 18–44 years, BRFSS 2014, *N* = 4858

Variables		OR (95% CI)		
		Discrimination at healthcare facilities	Discrimination at work	Emotional impact of discrimination
Sex (female)		0.82^NS^ (0.51, 1.31)	1.04^NS^ (0.70, 1.55)	1.20^NS^ (0.86, 1.70)
Age (references 18–24)	25–34	1.51^NS^ (0.78, 2.92)	0.76^NS^ (0.44, 1.31)	1.41^NS^ (0.85, 1.70)
	35–44	2.62^**^ (1.35, 5.13)	1.30^NS^ (0.78, 2.16)	1.89^*^ (1.13, 3.18)
Race	Black	2.45^**^ (1.24, 4.83)	6.24^***^ (3.71, 10.53)	5.05^***^ (3.32, 7.68)
	Hispanic	2.63^**^ (1.30, 5.33)	3.00^***^ (1.78, 5.08)	3.17^***^ (2.09, 4.81)
	Asian/other	1.65^NS^ (0.78, 3.49)	4.20^***^ (2.24, 7.88)	3.10^***^ (1.76, 5.49)
	Multiracial	7.28^**^ (1.94, 27.35)	6.94^**^ (2.04, 23.60)	9.36^***^ (3.55, 24.68)
Annual income (reference: lowest)	2nd lowest	0.93^NS^ (0.41, 2.08)	0.59^NS^ (0.30, 1.19)	1.13^NS^ (0.60, 2.13)
	Middle	0.58^NS^ (0.23, 1.43)	0.38^NS^ (0.17, 0.86)	0.62^NS^ (0.30, 1.29)
	2nd highest	0.81^NS^ (0.36, 1.83)	0.53^NS^ (0.25, 1.10)	1.09^NS^ (0.56, 2.12)
	Highest	0.25^**^ (0.10, 0.58)	0.49^NS^ (0.24, 1.02)	0.59^NS^ (0.31, 1.13)
Education (reference, < high school)	High school	1.07^NS^ (0.42, 2.72)	1.71^NS^ (0.87, 3.39)	1.29^NS^ (0.70, 2.36)
	Some college no degree	1.82^NS^ (0.72, 4.99)	1.11^NS^ (0.56, 2.22)	1.18^NS^ (0.66, 2.10)
	College graduate	1.11^NS^ (0.41, 2.99)	0.93^NS^ (0.45, 1.92)	1.24^NS^ (0.68, 2.28)

^***^p < 0.001, ^**^p < 0.01, ^*^p < 0.05, ^NS^ Not significant

All models are adjusted for race, age, sex, education, income

## Discussion

This study uniquely examined the association between three markers of racial discrimination, namely discrimination at healthcare facilities, discrimination at work and emotional impact of discrimination and tooth loss among American adults aged 18 to 44 years. While the three markers of discrimination were significantly associated with tooth loss, discrimination at healthcare facilities had the strongest association with tooth loss which persisted even after accounting for socioeconomic factors, health insurance and use of dental services. No known study has examined the relationship between discrimination and oral health. However, several studies demonstrated racial/ethnic inequality in tooth loss in the USA and other countries [4, 23–25]. Furthermore, studies have argued that socioeconomic inequalities explain ethnic variation in oral health [2, 26–28]. It is also possible that racial discrimination explains part of ethnic inequality in oral health. Discrimination could also indirectly influence oral health as it is linked to an individual’s socioeconomic condition which subsequently influences health-related behaviours and health outcomes [10, 11]. It is worth noting that in this study accounting for discrimination slightly attenuated the relationship between ethnicity and tooth loss but did not eliminate it.

While there are no studies on discrimination and oral health, there are several studies on discrimination and general health [12, 29, 30], which demonstrated an association between discrimination and poor general health.

Experience of discrimination impacts psychological wellbeing and causes distress. While this psychological impact can directly affect the body systems, it could also affect health-related behaviours. Individual experiencing psychological distress could adopt unhealthy behaviours such as smoking, excessive consumption of sugars and alcohol [31], which subsequently leads to periodontal disease and dental caries, and eventually tooth loss [24, 32]. The impact of discrimination, particularly discrimination at healthcare facilities could inhibit a person from using healthcare services particularly preventive service. This type of discrimination was clearly demonstrated in a study which showed the negative impacts of discrimination on the use of dental services [19].

Other ways by which discrimination could affect an individual is by limiting their access to better employment, education and selection of neighbourhood which increases their exposure to environmental risk factors and reduces their ability to adopt healthier lifestyle [12, 13]. This is mostly observed in individuals belonging to ethnic minority who are on lower income and are living in poor quality housing and deprived neighbourhood with poor access to health-promoting resources. Having demanding, routine, undesirable occupations or being unemployed for a longer period of time are also linked to discrimination on the one hand and poor health and health risk behaviours on the other [33, 34]. This type of discrimination and unfair treatment could lead to poorer health outcomes such as type 2 diabetes due to stress which could lead to obesity [15], hypertension [35] and other health conditions, including poor oral health.

The strengths of this study are in using a large nationally representative sample of American adults, assessing tooth loss among relatively younger population and accounting for several confounders for the relationship between racial discrimination and tooth loss. Furthermore, different indicators of discrimination were used. Finally, to the best of our knowledge, no other study has assessed this association.

There are few limitations of this analysis worth mentioning here. First, given that the exposure and the outcome were assessed simultaneously, temporality cannot be evaluated. Second, data used in this analysis was obtained from a subsample of total participants of BRFSS who completed questions related to the analysis. Third, self-reported data used in the survey are subjected to recall bias. Fourth, if a discriminatory occurrence is ambiguous, the one who experience discrimination may under or over report its incidence; hence, some subtle forms of discrimination may not be easily detected. Finally, due to data limitations, other sources of discrimination were not included. However, the three indicators of discrimination clearly showed association with tooth loss.

## Conclusion

This study distinctly exemplified that racial discrimination is associated with tooth loss among American adults aged 18 to 44 years. Three indicators of discrimination were significantly associated with tooth loss. However, after accounting for several covariates only discrimination at healthcare facilities remained significantly associated with tooth loss. Discrimination explained part of ethnic inequalities in oral health. The role of racial discrimination on oral health should be further explored in future research.

## Data Availability

Data are available from public access.
